# Enhanced efficacy of glycoengineered rice cell‐produced trastuzumab

**DOI:** 10.1111/pbi.14429

**Published:** 2024-07-17

**Authors:** Jun‐Hye Shin, Sera Oh, Mi‐Hwa Jang, Seok‐Yong Lee, Chanhong Min, Young‐Jae Eu, Hilal Begum, Jong‐Chan Kim, Gap Ryol Lee, Han‐Bin Oh, Matthew J. Paul, Julian K.‐C. Ma, Ho‐Shin Gwak, Hyewon Youn, Seong‐Ryong Kim

**Affiliations:** ^1^ Department of Life Science Sogang University Seoul South Korea; ^2^ PhytoMab Co. Ltd. Seoul South Korea; ^3^ Department of Nuclear Medicine, Cancer Imaging Center Seoul National University Hospital Seoul South Korea; ^4^ Cancer Research Institute, Seoul National University College of Medicine Seoul South Korea; ^5^ Department of Chemistry Sogang University Seoul South Korea; ^6^ Hotung Molecular Immunology Unit, Institute for Infection and Immunity St George's University of London London UK; ^7^ National Cancer Center Korea Goyang‐si, Kyunggi‐do South Korea

**Keywords:** rice, glycoengineering, suspension cell, PhytoRice, trastuzumab, P‐TMab

## Abstract

For several decades, a plant‐based expression system has been proposed as an alternative platform for the production of biopharmaceuticals including therapeutic monoclonal antibodies (mAbs), but the immunogenicity concerns associated with plant‐specific N‐glycans attached in plant‐based biopharmaceuticals has not been completely solved. To eliminate all plant‐specific N‐glycan structure, eight genes involved in plant‐specific N‐glycosylation were mutated in rice (*Oryza sativa*) using the CRISPR/Cas9 system. The glycoengineered cell lines, PhytoRice®, contained a predominant GnGn (G0) glycoform. The gene for codon‐optimized trastuzumab (TMab) was then introduced into PhytoRice® through *Agrobacterium* co‐cultivation. Selected cell lines were suspension cultured, and TMab secreted from cells was purified from the cultured media. The amino acid sequence of the TMab produced by PhytoRice® (P‐TMab) was identical to that of TMab. The inhibitory effect of P‐TMab on the proliferation of the BT‐474 cancer cell line was significantly enhanced at concentrations above 1 μg/mL (****P < 0.0001). P‐TMab bound to a FcγRIIIa variant, FcγRIIIa‐F158, more than 2.7 times more effectively than TMab. The ADCC efficacy of P‐TMab against Jurkat cells was 2.6 times higher than that of TMab in an *in vitro* ADCC assay. Furthermore, P‐TMab demonstrated efficient tumour uptake with less liver uptake compared to TMab in a xenograft assay using the BT‐474 mouse model. These results suggest that the glycoengineered PhytoRice® could be an alternative platform for mAb production compared to current CHO cells, and P‐TMab has a novel and enhanced efficacy compared to TMab.

## Introduction

Monoclonal antibodies (mAbs) have become the dominant class of newly developed drugs and the best‐selling drugs worldwide in the pharmaceutical market since the first mAb was approved by the United States Food and Drug Administration (US FDA) in 1986 (Ecker *et al*., 2015; Lu *et al*., 2020). To date, most therapeutic mAbs are known to target cancer cells and autoimmune diseases (Stelter *et al*., 2020). Of these, TMab is a well‐known blockbuster mAb widely used for the treatment of breast and gastric cancer. Currently, mAb therapeutics are mostly produced using Chinese Hamster Ovary (CHO) cell cultures, which have high productivity. However, there are several issues, such as the high cost of the cell culture media and the risk of contamination with pathogens that cause zoonotic diseases, leading to high production costs (Shanmugaraj *et al*., 2020).

Breast cancer has been reported as the most commonly occurring cancer in women, causing more than 500 000 deaths worldwide per year (Komarova *et al*., 2019). Human epidermal growth factor receptor 2 (HER2)‐positive breast cancer, which affects approximately 20%–30% of all breast cancer patients, is characterized by the overproduction of HER2 (Komarova *et al*., 2019). The HER2 protein in breast and gastric cancer cells has been identified as a validated therapeutic target, as demonstrated in the functional study of TMab (Bang *et al*., 2010; Cobleigh *et al*., 1999; Slamon *et al*., 2001). Currently, the combined use of chemotherapy and TMab is considered the primary medical treatment for HER2‐positive breast cancer (Hudis, 2007). However, its effectiveness is limited in treating metastatic HER2‐positive breast cancer and metastatic breast cancer with low HER2 expression (Komarova *et al*., 2019).

The modes of action of TMab are categorized into non‐immune and immune‐mediated mechanisms (Musolino *et al*., 2022). The non‐immune mechanism results from the binding of antibody fragment antigen‐binding (Fab) domains to HER2 receptors, leading to the anti‐proliferative effects of cancer cells by perturbing HER2‐signalling (Slamon *et al*., 2001). The immune response is initiated by the physical interaction of the fragment crystallizable (Fc) domains of tumour cell‐bound antibodies with Fc receptors (FcRs) on the surface of immune cells. This interaction leads to the elimination of cancer cells through effector functions such as antibody‐dependent cellular cytotoxicity (ADCC) or antibody‐dependent cellular phagocytosis (ADCP), which are the primary functions of antibody therapeutics (Arnould *et al*., 2006; Gül and van Egmond, 2015). The immune‐mediated mechanism of antibody therapeutics relies on N‐glycosylation, which plays an important role in affecting the binding affinity to FcRs of immune cells. While normal fucosylated TMab demonstrated a significant reduction in effector functions such as ADCC (Arnould *et al*., 2006), afucosylated TMab exhibited significantly improved ADCC effector efficacy, resulting in superior suppression of cancer cell growth (Fiedler *et al*., 2018; Junttila *et al*., 2010; Suzuki *et al*., 2007). Similarly, the recently approved margetuximab has also demonstrated enhanced binding affinity to FcγRIIIa (CD16A) and reduced binding affinity to FcγRIIb (CD32B), thereby improving ADCC function (Nordstrom *et al*., 2011; Rugo *et al*., 2021). These results strongly indicate the potential for developing a biobetter by enhancing the effector function of the Fc domain.

For a long time, alternative platforms to CHO‐based platforms have been sought, and the plant‐based platform has gained attention due to its merits, such as being free from zoonotic microorganisms and cost‐effectiveness (Chen and Davis, 2016). Thus, the plant cell‐based Elelyso® (enzyme therapeutics for Gaucher's disease) and Elfabrio® (enzyme therapeutics for Fabry disease), as well as the tobacco leaf‐based Covifenz® (COVID‐19 vaccine), have been approved to date (Fox, 2012; Hughes *et al*., 2023; Mirasol, 2022; Ruocco and Strasser, 2022).

For the development of plant‐based mAb therapeutics, consideration should be given to the post‐translational modification (PTM) of the mAb protein, as it is influenced by plant‐specific N‐glycosylation (Castilho and Steinkellner, 2012). The plant‐specific N‐glycans may cause immunogenicity in humans (Gomord *et al*., 2010; Santos *et al*., 2016). Various studies using RNAi or CRISPR/Cas9 have been conducted to reduce or eliminate plant‐specific N‐glycans (Cox *et al*., 2006; Göritzer *et al*., 2022; Hanania *et al*., 2017; Herman *et al*., 2023; Mercx *et al*., 2017; Shin *et al*., 2011; Stelter *et al*., 2020). However, there are no reports of complete elimination of plant‐specific N‐glycans.

In this study, rice cell lines have been developed to completely remove plant‐specific N‐glycans using CRISPR‐Cas9‐based GlycoPhyto technology, which includes Fc glycoengineering and repeated selection of cell lines with Sanger sequencing. The established PhytoRice® cell lines exhibited highly uniform GnGn (G0) N‐glycan in the secreted proteins, as well as in the total soluble proteins. Using the PhytoRice® cell line, a TMab was successfully produced, purified, and named P‐TMab. P‐TMab specifically binds to the HER2 receptor with enhanced binding affinity. Furthermore, the binding affinity to FcγIIIa variants and the ADCC activity of P‐TMab were significantly improved compared to those of TMab. This suggests that P‐TMab has the potential to be a biobetter, enhancing the effector function of the Fc domain.

## Results

### Establishment of glycoengineered rice cell lines

*β1,2‐Xylosyltransferase* (*β1,2‐XylT*) and *α1,3‐Fucosyltransferase* (*α1,3‐FucT*) are known to exist as single genes in the rice genome (Harmoko *et al*., 2016; Jung *et al*., 2021; Takano *et al*., 2015). In addition, a rice *β1,3‐Galactosyltransferase* (*β1,3‐GalT*) gene was recently elucidated (Jung and Kim, 2023). However, there are no reports about other genes involved in plant‐specific N‐glycosylation, such as *α1,4‐Fucosyltransferase* (*α1,4‐FucT*) and *hexosaminidase* (*HEXO*) genes, in the rice. To identify candidate genes in rice, a BLAST search was conducted using the amino acid sequences of orthologous genes confirmed in Arabidopsis (Liebminger *et al*., 2011; Strasser *et al*., 2007). In the results, LOC_Os12g07290, LOC_Os05g02510 and LOC_Os03g11980 were identified as putative *α1,4‐FucT, HEXO1* and *HEXO2* genes, respectively (Table S1). Interestingly, both LOC_Os01g66700 and LOC_Os05g34320 were identified as *OsHEXO3* candidates, which is unlike *Arabidopsis* and *Nicotiana benthamiana*, which have a single *HEXO3* gene (Liebminger *et al*., 2011; Shin *et al*., 2017; Strasser *et al*., 2007). Thus, four *HEXO* gene candidates (LOC_Os05g02510 as *HEXO1*, LOC_Os03g11980 as *HEXO2*, LOC_Os01g66700 as *HEXO3* and LOC_Os05g34320 as *HEXO4*) were ultimately identified in the rice genome (Table S1).

**Figure 1:**
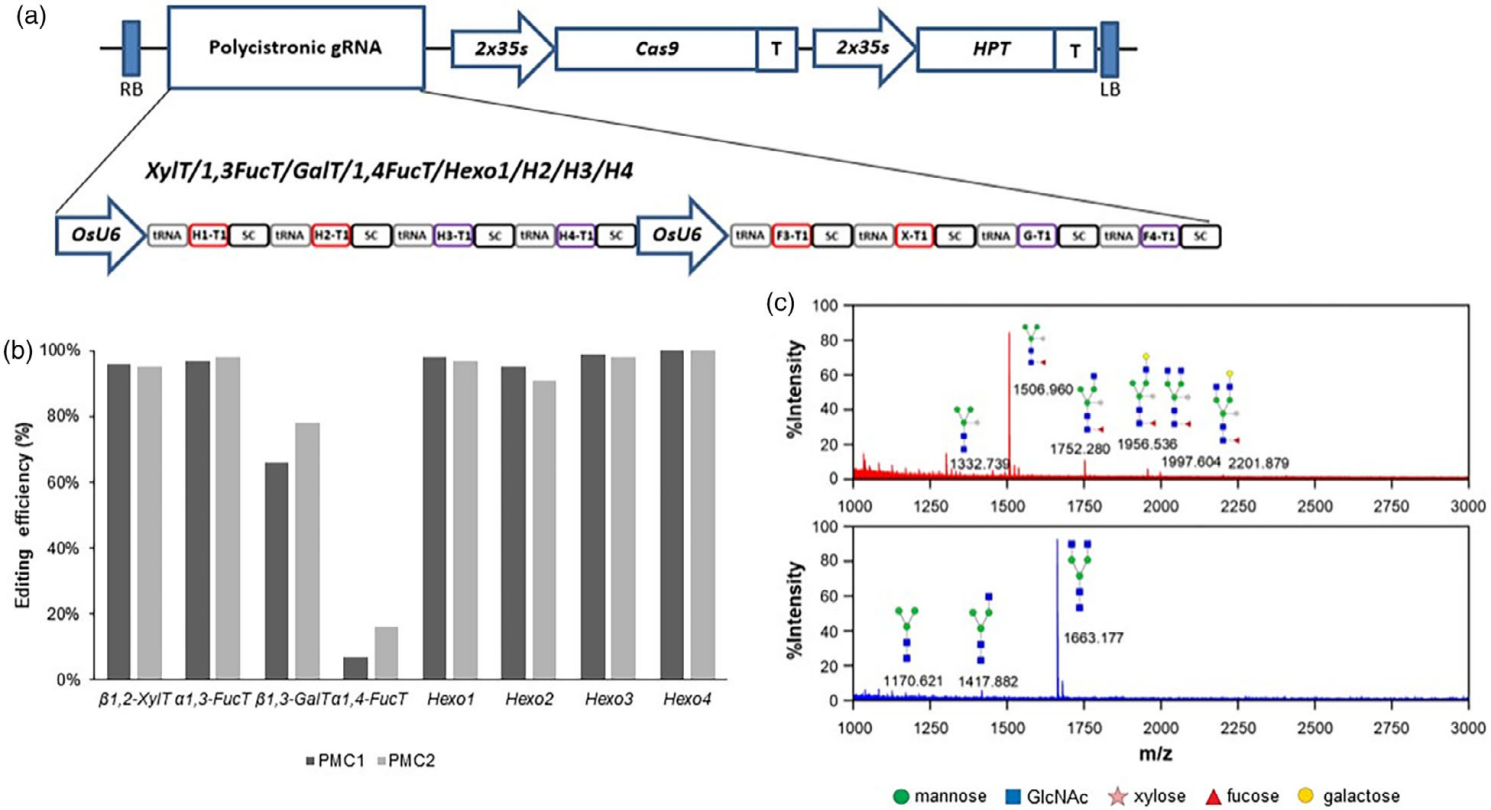
Construction and characterization of CRISPR‐Cas9‐based glycoengineered rice cell lines. (a) The diagram of pPM101. The multiplex CRISPR‐Cas9 vector that expresses Polycistronic single guide RNAs (sgRNAs) targeted into eight genes involved in plant‐specific N‐glycosylation pathway was constructed. The two expression cassettes of polycistronic sgRNA sequences driven by rice U6 promoter were inserted into CRISPR‐Cas9 vector, named pPM101. The detailed information of sgRNAs used was summarized in Table S1. (b) INDEL efficiencies occurred in eight genes from final selected cell lines. pPM101 was integrated into rice genome via *Agrobacterium*‐mediated transformation method. The cells (PMC1 and 2) were finally selected using callus line showing the highest INDEL efficiency. The efficiency of genome editing was analysed by ICE tool (https://ice.synthego.com/#/). (c) N‐glycan profile of PMC1, named PhytoRice®. The N‐glycan structures purified from total secreted proteins were analysed using MALDI‐TOF. In PMC1, GnGn form was detected, indicating entire elimination of plant‐specific N‐glycan (Bottom panel), whereas various N‐glycan structures containing plant‐specific sugar such as β1,2‐xylose, α1,3 or α1,4‐fucose and β1,3‐galactose were found in WT (Upper panel). Each symbol represents a specific monosaccharide (green circle, mannose; square, GlcNAc; star, xylose; triangle, fucose; yellow circle, galactose).

To knock out a total of eight genes (*β1,2‐XylT, α1,3‐FucT, β1,3‐GalT, α1,4‐FucT* and four putative *HEXO* genes) simultaneously, one specific target sequence per gene was selected using the CRISPR‐P gRNA design tool (Liu *et al*., 2017). As shown in Table S1, all selected target sequences were located in the 5' region of the coding sequence of each gene (Exon 1 or 2) to increase the possibility of causing a nonsense mutation via insertion or deletion (INDEL). Two expression cassettes containing polycistronic gRNA sequences, each consisting of four target sequences, driven by the rice U6 promoter, were synthesized and inserted into a CRISPR‐Cas9 vector, resulting in the generation of pPM101 (Figure 1a). After rice callus transformation mediated by *Agrobacterium* carrying pPM101, hygromycin‐resistant calli were selected, and the genomic regions around the target sequence were amplified using PCR. The PCR products were sequenced and then analysed using ICE analysis available at https://ice.synthego.com/#/. After several rounds of repeated selection, the callus line #1‐12‐20‐11 was ultimately chosen for its editing efficiency (Figure S1a), and confirmed by immunoblotting using anti‐β1,2‐XylT and anti‐α1,3‐FucT antibodies (Figure S1b). As indicated in Figure S1a, the gene editing efficiency was nearly 100% for all genes except for *β1,3‐GalT* (57%) and *α1,4‐FucT* (23%). The callus from line #1‐12‐20‐11 was further divided into single‐cell clones after suspension culture and microscopic observation were conducted. Finally, two independent cell lines, PhytoMab‐producing Cell (PMC)1 and PMC2, were selected based on the extent of genome editing and their N‐glycan profile (Figure 1b). The PMC1 and PMC2 calli showed a tendency to grow normally, but they were very fragile and fine compared to the wild‐type callus (Figure S1c). In addition, plant regeneration failed in the KO cells, a phenomenon consistent with previous reports on the knockout of genes associated with N‐glycosylation (Fanata *et al*., 2013; Jung *et al*., 2021).

### All plant‐specific N‐glycan was completely eliminated in the glycoengineered cell lines

For the PMC lines, the N‐glycans of proteins and the loci of T‐DNA insertion were examined. The N‐glycans of secreted proteins in the spent media, as well as the total soluble proteins of the suspension cells, were analysed using mass spectrometry (MS). For the PMC1 cell line, the proportion of GnGn was 97.4% for the secreted proteins, compared to 80.8% for the total soluble proteins (Figure 1c and Table S2). In PMC2, the proportion of GnGn in total soluble proteins and secreted proteins was 92.3% and 81.9%, respectively (Table S2). These results indicate that the plant‐specific N‐glycans were eliminated in the cell lines (Figure 1c and Table S2).

### The number of pPM101 T‐DNA insertions into genome of PMCs

For future use of the PMC cell lines, the characteristics of the cells should be identified. Therefore, the using inverse PCR. For the PMC1 cell line, T‐DNA was solely found in the 4th intron of LOC_Os01g29409 on chromosome 1 (Figure S2a). For the PMC2 line, T‐DNAs were identified in LOC_Os01g29409 and the intergenic region between LOC_Os02g44780 and LOC_Os02g44810, respectively (Figure S2b). No correlation between the number of T‐DNA insertions and genome editing efficiency was observed. The genome‐edited N‐glycan‐engineered cells were named and registered as PhytoRice. The PMC1 line was further used for the production of TMab.

### Expression of TMab in PhytoRice®

**Figure 2:**
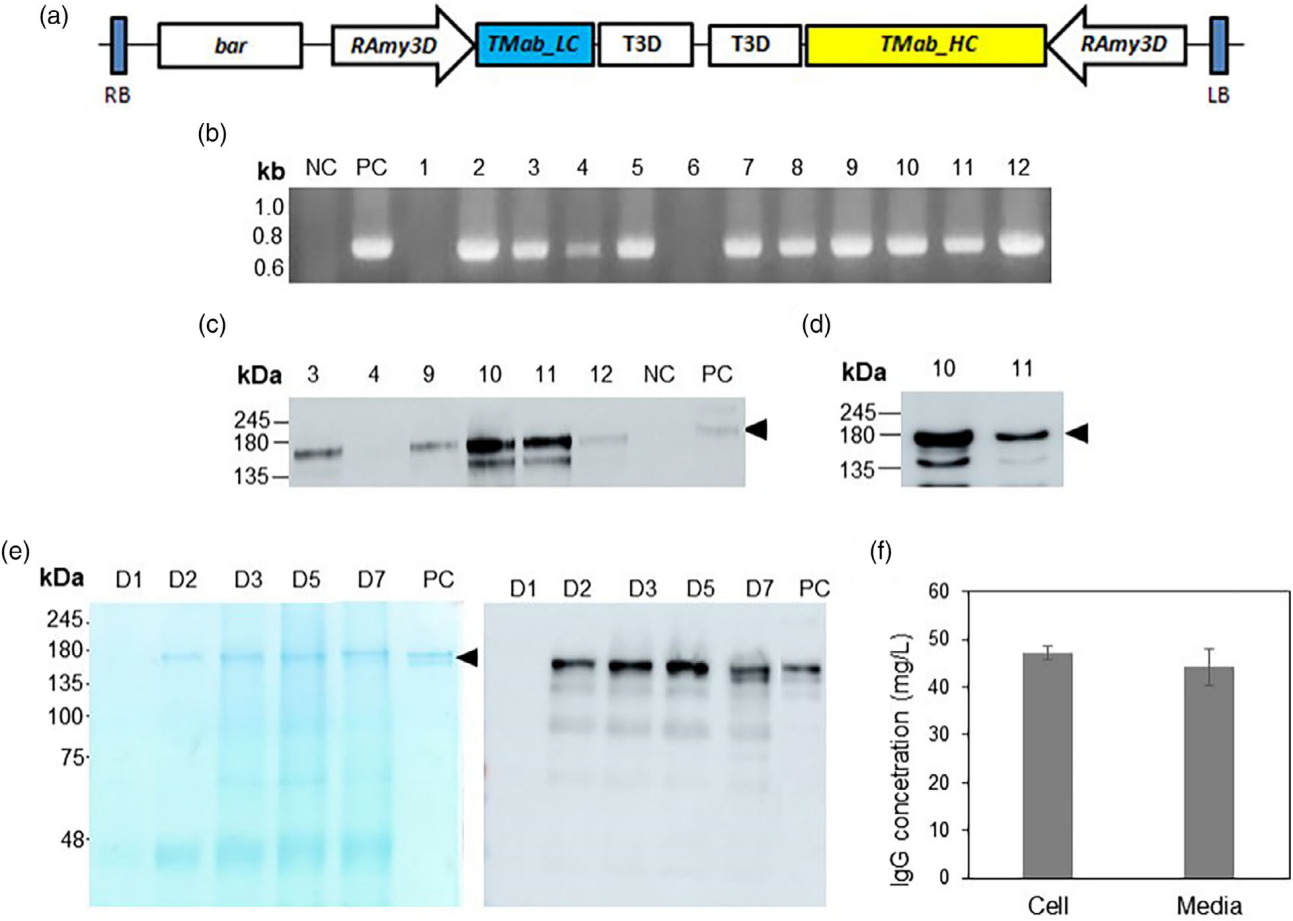
Construction of TMab expression vector and screening of PhytoRice®‐based TMab‐expressing rice cell lines. (a) The diagram of pPM102 expressing TMab. Codon‐optimized TMab light chain (LC) and heavy chain (HC) genes were inserted into separate expression cassettes driven by the rice amylase 3D (*RAmy3D*) gene promoter, resulting in the construction of the TMab LC and HC co‐expression vector, pPM102. (b) Genomic DNA (gDNA) PCR using the primer set for the TMab‐LC gene. Approximately 700 base pair‐sized bands were detected in phosphinothricin (PPT)‐resistant callus lines. NC, WT callus (cv. Dongjin); PC, pPM102 plasmid; lane 1–12, PPT‐resistant callus lines. (c) Immunoblotting of transgenic callus lines selected using gDNA PCR. The calli were cultured in induction media without sucrose for 7 days. Afterwards, cell lysate was prepared and separated under non‐reducing conditions in SDS/PAGE. The TMab was detected using rabbit α‐human IgG‐HRP at a dilution factor 1 : 5000. The arrowhead indicates a TMab with a size of 150 kDa. NC, WT callus (Dongjin); PC, TMab (5 ng were loaded). (d) Immunoblotting of two cell lines (#10 and #11) selected based on the expression level. The suspension cells were incubated with induction media without sucrose for 7 days. Equal amount (10 μg) of total secreted proteins obtained from the cultured media were separated under non‐reducing conditions using a 7.5% SDS/PAGE, and immunoblotting was performed using rabbit α‐human IgG‐HRP at a dilution factor 1 : 5000. (e) The induction pattern of TMab using a secretory‐Phyto101 suspension cell expression system. Cells well‐grown under suspension cultivation were inoculated into induction liquid media and then incubated for 7 days. The spent media sample was separated under non‐reducing conditions in a 7.5% SDS/PAGE (left panel), and immunoblotting was performed using rabbit anti‐human IgG‐HRP (right panel). D, days after induction; arrowhead, TMab; PC, TMab. (e, f) The quantification of TMab induced in Phyto101. ELISA was performed using cells induced for 5 days and their spent media. The standard deviation was calculated using the values from two biological replicates.

To produce TMab immunoglobulin in PhytoRice®, both the heavy chain and light chain of TMab were codon‐optimized, synthesized, and then cloned into a binary vector under the *Ramy3D* promoter, resulting in the creation of pPM102 (Figure 2a). Using *Agrobacterium tumefaciens* LBA4404, T‐DNA region of pPM102 was introduced into PMC1 line belong to PhytoRice®. Transgenic calli were selected on agar plates containing phosphinothricin (PPT), and genomic DNA PCR was conducted using primers for the light chain gene of TMab. As shown in Figure 2b, the expected 0.7 kb‐sized bands were detected in the calli, and those positive calli were subjected to western blotting using a human IgG antibody. About 150 kDa bands corresponding to the size of TMab were detected on the non‐reducing SDS‐PAGE (Figure 2c). The expression level of TMab in each transgenic callus varied, and among the calli examined, lines #10 and #11 were selected for suspension cell culture.

In addition, we introduced T‐DNA of pSK446 (Figure S3a) to wild‐type rice calli via *Agrobacterium* and then obtained transgenic callus lines that express TMab (Figure S3a,b). Of them, WT‐#44 was used for further study (Figure S3c,d).

### Establishment of a TMab‐producing cell line

Among lines #10 and #11, line #10 exhibited a higher expression of TMab in the suspension culture (Figure 2d) and was designated as PHYTO101. The PHYTO101 callus was suspended in N6 liquid media containing 2 mg/L of 2,4‐D and 0.02 mg/L of kinetin. After establishing the suspension cell culture, PHYTO101 cells that had been grown for 7 days in the growth media were transferred to media without sucrose and then incubated for an additional 7 days. TMab expression was detected in the spent media after Day 2 and reached its highest level on Day 5 (Figure 2e).

To quantify the amount of TMab produced on Day 5, an enzyme‐linked immunosorbent assay (ELISA) was performed using the cells and the spent media from Day 5. Similar levels of TMab were detected in both the cells and the media, at 47.2 and 44.3 mg/L, respectively (Figure 2f). Therefore, the productivity of TMab in Phyto101 was approximately 91.5 mg/L.

### Sequence and conformational analyses of PHYTO101‐produced TMab showed high similarity to those of TMab

**Figure 3:**
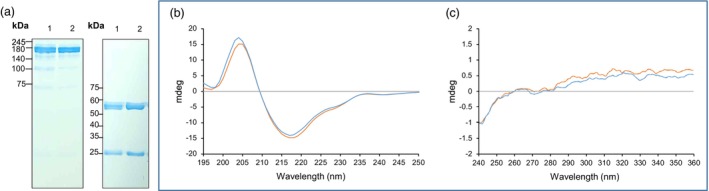
Purification and structural characterization of P‐TMab from Phyto101. (a) P‐TMab was purified using a 3‐step method. P‐TMab produced in the Phyto101 platform was sequentially purified using Protein A, anion exchange, and hydroxyapatite column chromatography. The purified P‐TMab was separated under non‐reducing (left panel) and reducing (right panel) conditions using a 7%–15% gradient SDS/PAGE. Lane 1, P‐TMab; Lane 2, TMab. (b, c) The conformational characteristics of P‐TMab were examined using Circular Dichroism (CD) analysis. The secondary (b) and tertiary (c) structures of P‐TMab and TMab were determined by Far‐UV (195–250 nm) and near‐UV (250–350 nm) CD absorption spectra.

The TMab produced from the PHYTO101 cell line was purified using chromatography and named P‐TMab. As shown in Figure 3a, a 150 kDa band was observed under non‐reduced conditions, while bands of 50 and 25 kDa were observed under reduced conditions. To confirm whether the amino acid sequence of P‐TMab is the same as that of TMab, full‐length amino acid sequencing was performed using LC/MS/MS analysis of trypsin‐ or Glu‐C‐treated heavy and light chains of P‐TMab and TMab. As shown in Table S3, the sequences of P‐TMab separated by LC/MS were 100% matched with those of TMab at the peptide level. Following additional LC/MS/MS analysis, it was determined that the total coverages for the amino acid sequences of TMab and P‐TMab were up to 94% and 93.7%, respectively. It was also observed that the amino acid sequence of P‐TMab obtained by the analysis was 100% identical to that of TMab (Table S4).

The secondary and tertiary structures of P‐TMab were compared with those of TMab using circular dichroism (CD). As depicted in Figure 3b, the Far‐UV CD absorption spectra, measured in the range from 195 to 250 nm, exhibited a similar pattern between P‐TMab and TMab. They displayed the characteristic features of proteins with high β‐sheet content, including a positive band around 202 nm and a negative band at approximately 218 nm (Moro Pérez *et al*., 2019). The near‐UV CD spectra of P‐TMab obtained in the 250‐350 nm interval also showed similarity to that of TMab (Figure 3c), indicating that P‐TMab has a similar structure to TMab in its native condition.

### P‐TMab exhibits almost homogeneous N‐glycan profile

**Figure 4:**
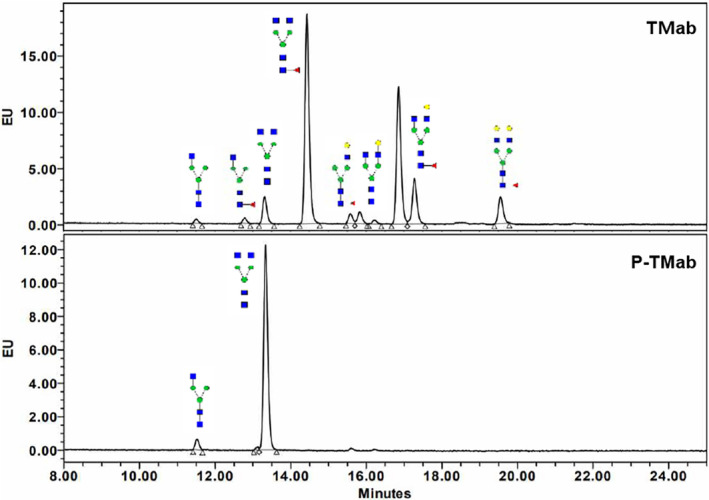
The analysis of N‐glycan attached to P‐TMab. N‐glycans purified from P‐TMab and TMab were separated using the UPLC‐FLD method. The GnGn (G0) form was exclusively found in the P‐TMab (bottom panel), while heterogeneous N‐glycan forms were detected in the TMab (upper panel).

TMab is well‐known to contain N‐glycan linked to the asparagine residue at position 300 (ASN300) in the CH2 domains (Nebija *et al*., 2014). N‐glycan analysis of P‐TMab using the UPLC‐FLD method revealed that the GnGn (G0) form was exclusively present in P‐TMab, while heterogeneous N‐glycan forms were detected in TMab (Figure 4 and Table S5). This indicates the complete elimination of plant‐specific N‐glycan in P‐TMab. Moreover, 90.5% of the N‐glycan found in TMab consists of various fucosylated forms, with only 9.5% in the afucosylated form (G0‐GN, G0, and G1 at 0.7%, 5.4%, and 3.4%, respectively). In contrast, all N‐glycan found in P‐TMab was in non‐fucosylated form, such as G0 and G0‐GN, which accounted for 95.5% and 4.5%, respectively (Table S5).

### P‐TMab bound HER2 to inhibit the proliferation of BT‐474 cells

**Figure 5:**
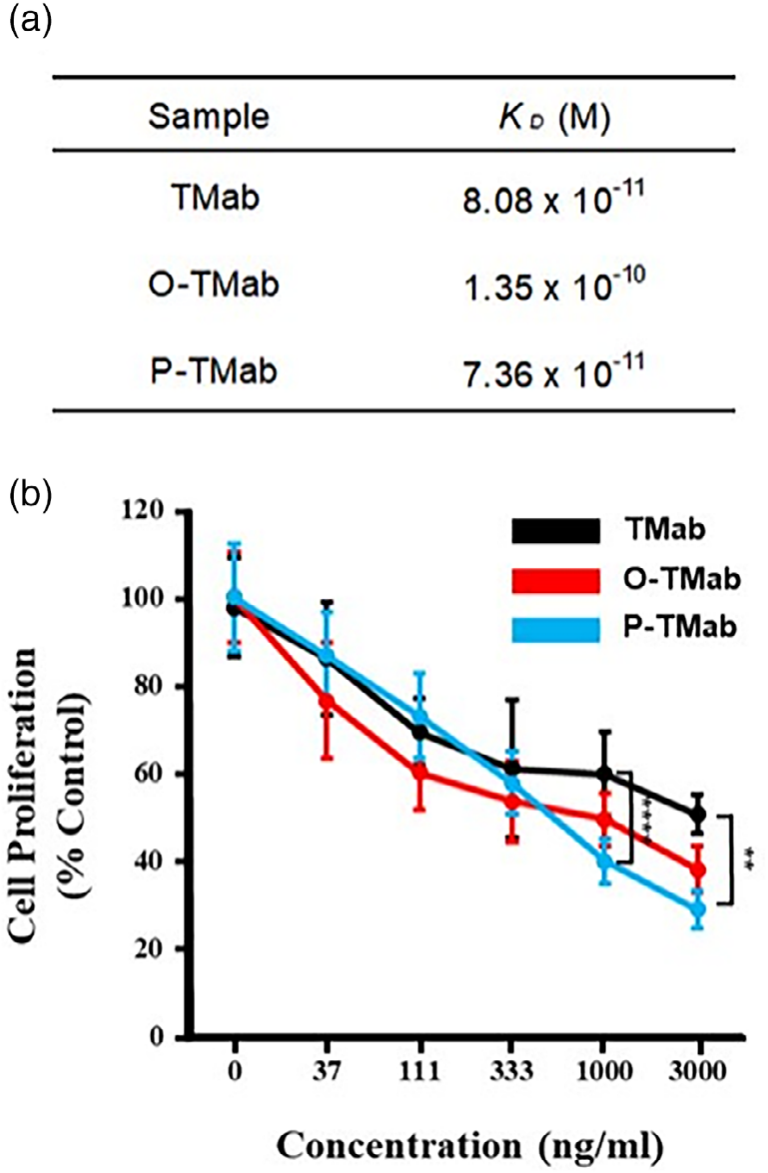
HER2‐binding affinity and HER2‐specific anti‐proliferative effect of P‐TMab. (a) Bio‐layer interferometry (BLI) was used to measure and compare the binding affinity to HER2 among O‐TMab (produced from transgenic wild‐type rice), P‐TMab and TMab. (b) CCK‐8 assay. CCK‐8 solution was added after BT‐474 cells treated with O‐TMab, P‐TMab or TMab were incubated for 72 h. The data in this study are depicted as mean values from three biological replicates, accompanied by standard errors of the mean (SEM) indicated as ±. Statistical significance was assessed through unpaired Student's *t*‐tests. **P < 0.01; ****P < 0.0001.

TMab blocks HER2‐mediated cell signalling by binding to HER2, thereby inhibiting cancer cell proliferation (Musolino *et al*., 2022). The degree of binding affinity to HER2 correlates with its functional activity. We utilized Bio‐layer interferometry (BLI) to measure the binding affinity and confirm the physical interaction between P‐TMab and HER2. As shown in Figure 5a, the equilibrium dissociation constant (*K*_D_) values of P‐TMab and TMab were 0.07 nM and 0.08 nM, respectively. This indicates a slightly higher binding affinity of P‐TMab to HER2 compared to TMab. Moreover, O‐TMab, produced from transgenic wild‐type rice, exhibited a reduced binding affinity to HER2 with a *K*_D_ value of 0.13 nM (Figure 5a).

We investigated the anti‐proliferative effect of P‐TMab on breast cancer cells *in vitro*. As shown in Figure 5b, P‐TMab inhibited the growth of BT‐474 cells. The anti‐proliferative effect of P‐TMab was similar to that of TMab at a lower concentration of 1 μg/mL. However, at concentrations higher than that, the anti‐proliferative effect of P‐TMab was more pronounced than that of TMab (Figure 5b). O‐TMab exhibited a similar anti‐proliferative effect to that of TMab at all concentrations tested (Figure 5b). Taken together, the results indicate that both P‐TMab and O‐TMab have the same mechanism of action as TMab, and P‐TMab exhibits an enhanced anti‐tumour effect.

### Binding affinity of P‐TMab to FcγRIIIa variants was significantly enhanced

The immune‐mediated mechanism of antibody therapeutics is well known to involve N‐glycosylation, which impacts the binding affinity to Fcγ receptors on immune cells. Two common alleles of the FcγRIIIa (CD16a) gene encode two variants that differ at position 158, either valine (V158) or phenylalanine (F158). It is known that FcγRIIIa‐F158, which is a major allele of FcγRIIIa, shows approximately 10‐fold less binding affinity to IgG1 than FcγRIIIa‐V158 (Koene *et al*., 1997; Pereira *et al*., 2018).

**Figure 6:**
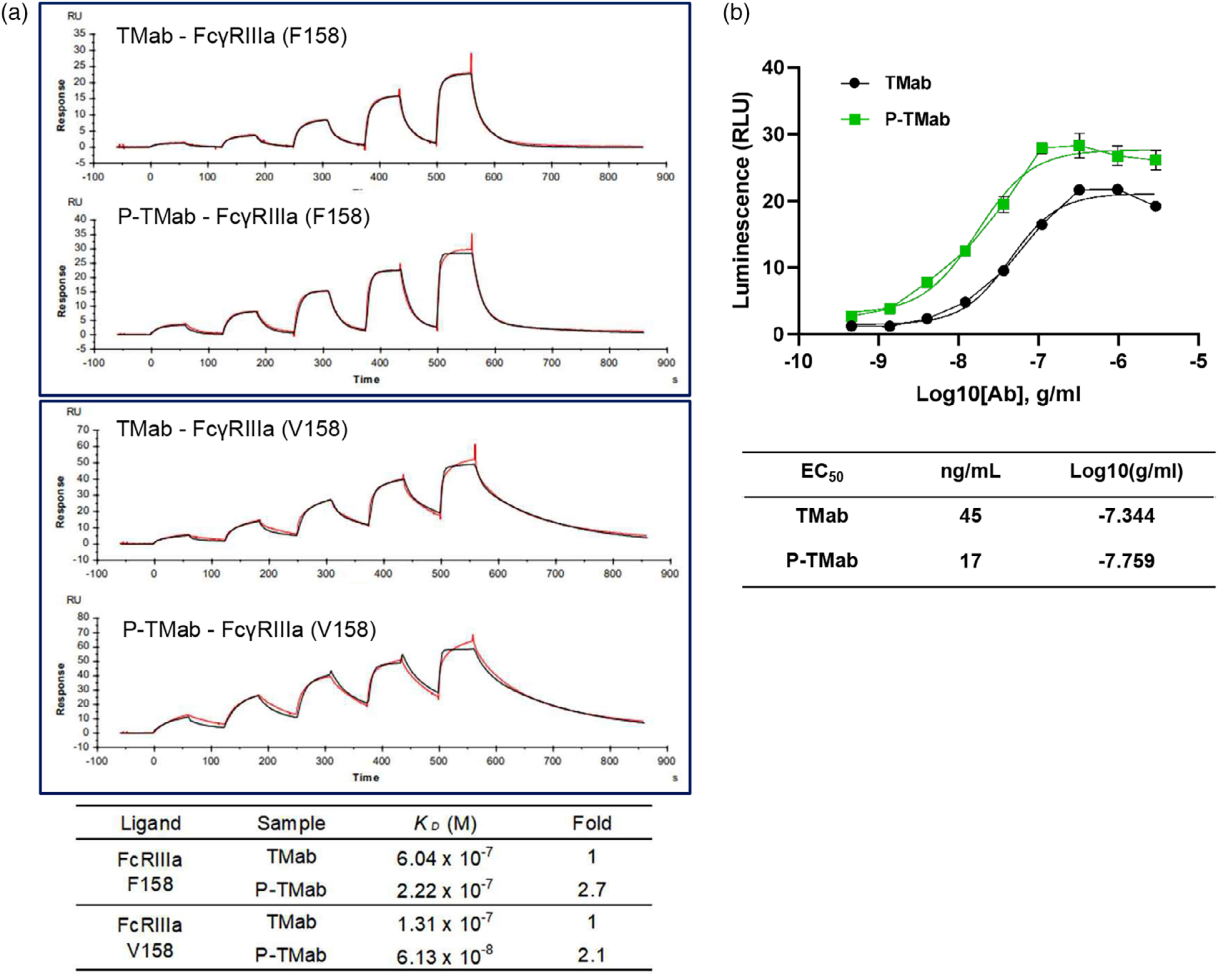
The binding affinity of P‐TMab to FcγRIIIa variants and *in vitro* ADCC assay. (a) Surface plasmon resonance (SPR) assay was used for checking out the binding affinity of TMab and P‐TMab to FcγRIIIa‐F158 (upper box) and FcγRIIIa‐V158 (middle box). Bottom panel is a summary of the SPR assay. (b) *in vitro* ADCC assay. BT474 cells treated with TMab or P‐TMab were incubated for 24 h after the treatment with Jurkat cells. The data in this study are depicted as mean values from three biological replicates, accompanied by standard errors of the mean (SEM) indicated as ±.

The binding affinity of P‐TMab to FcγRIIIa variants was assessed using Surface Plasmon Resonance (SPR). Whereas the *K*_D_ of TMab to the FcγRIIIa‐F158 was 604 nM, the *K*_D_ value of P‐TMab was 222 nM. This indicates that the binding affinity of P‐TMab to FcγRIIIa‐F158 is 2.7 times higher than that of TMab (Figure 6a). The *K*_D_ value of P‐TMab and TMab to FcγRIIIa‐V158 were 61 nM and 130 nM, respectively (Figure 6a). These results suggest that P‐TMab could enhance the anti‐cancer effectiveness through improved Fc effector function.

### Enhanced ADCC efficacy of P‐TMab *in vitro*

To investigate whether P‐TMab enhances ADCC efficacy, we conducted an *in vitro* ADCC assay. As shown in Figure 6b, the intensity of luminescence tends to increase as the amount of both mAbs is increased. Notably, under all the examined concentration conditions, cells treated with P‐TMab exhibited significantly higher luminescence compared to cells treated with TMab (Figure 6b). The EC50 values for P‐TMab and TMab were 17 ng/mL and 45 ng/mL, respectively (Figure 6b). In contrast, the luminescence intensity was significantly lower in O‐TMab‐treated cells than in TMab‐treated cells (Figure S4). The results strongly indicate that P‐TMab outperforms TMab in terms of ADCC efficacy, primarily by enhancing its binding affinity to FcγRIIIa.

### P‐TMab showed efficient tumour targeting but had less liver uptake than TMab

**Figure 7:**
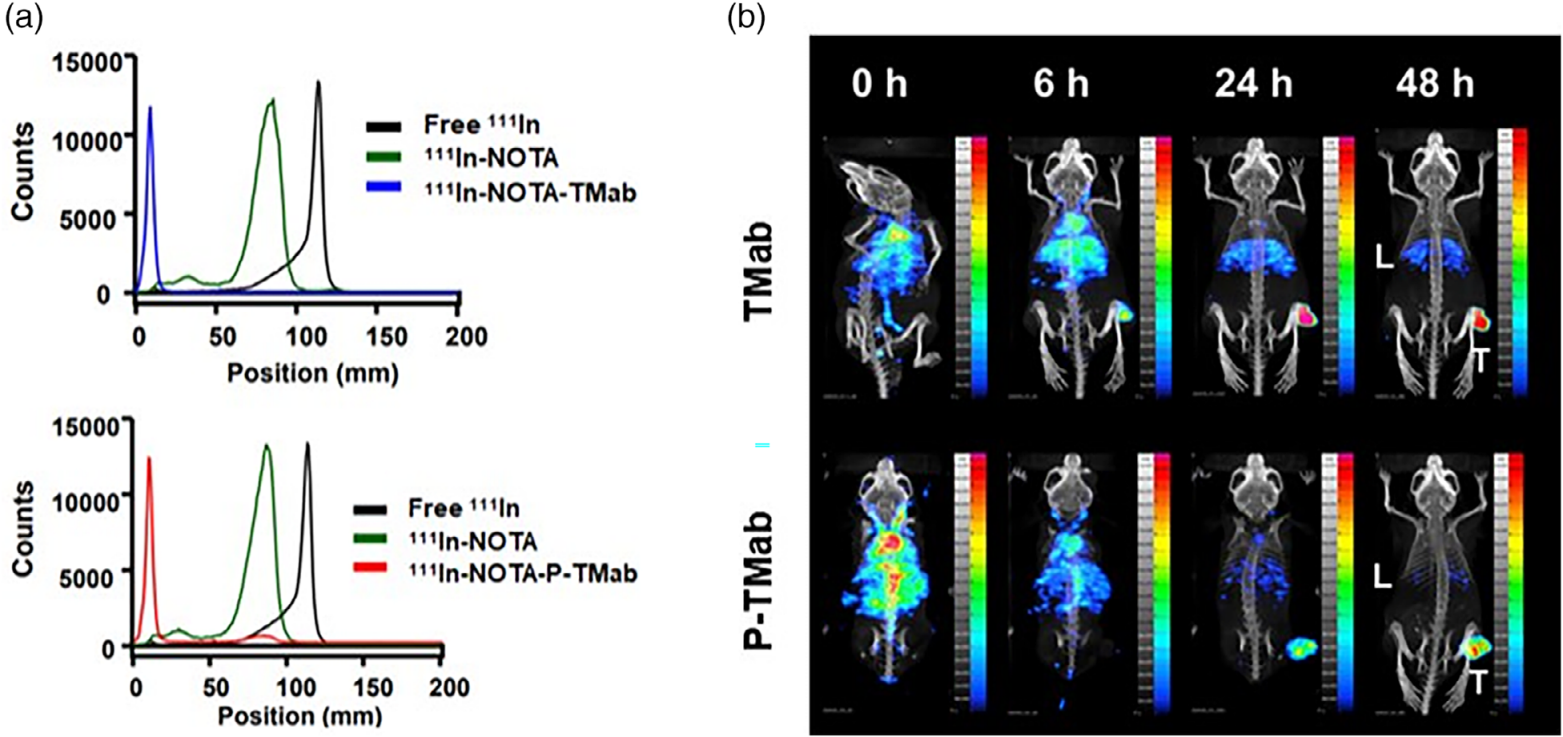
*In vivo* biodistribution pattern of P‐TMab. (a) TLC results confirm antibody labelling efficiency of the radioactive isotope ^111^Indium. (b) SPECT/CT images of ^111^In‐labelled antibodies in BT474 tumour model. L and T indicate liver and tumour, respectively.

To examine the *in vivo* distribution of P‐TMab, we conducted SPECT/CT imaging using ^111^Indium‐labelled antibodies in a HER2‐positive BT‐474 tumour model. After labelling the antibody with the radioactive isotope ^111^Indium, the efficiency of the labelling process was determined using thin‐layer chromatography. The results showed that the labelling efficiency was almost equal for both antibodies (Figure 7a). ^111^In‐labelled TMab or P‐TMab antibodies were administered via tail vein injection to mice bearing BT‐474 tumours. The results indicated a significant increase in antibody uptake in the tumour for both P‐TMab and TMab‐treated mice for up to 48 h after antibody injection (Figure 7b). Remarkably, the uptake pattern of P‐TMab in the liver differed from that of TMab. P‐TMab exhibited reduced liver uptake compared to TMab starting from 6 h after antibody injection. Furthermore, P‐TMab was scarcely detected in the liver after 48 h, while TMab was still detectable in the liver at the same time point (Figure 7b). The results suggest that P‐TMab can be targeted efficiently to tumours and may also reduce potential liver toxicity compared to TMab.

## Discussion

Because nearly all therapeutic proteins undergo glycosylation, it is important to utilize a platform capable of conducting post‐translational modification (Dammen‐Brower *et al*., 2022). It is well‐known that plants undergo a post‐translational modification process similar to that of mammals. Therefore, plant‐based platforms are emerging as alternative platforms to animal cell‐based platforms for this reason. In addition, the plant‐based manufacturing system is expected to reduce production and purification costs compared to the animal cell culture system, due to the lower cost of plant culture medium (Doran, 2006; Kwon *et al*., 2009). Several techno‐economic analyses have shown that plant‐based systems could be competitive with the current CHO cell culture system (Buyel *et al*., 2017; Corbin *et al*., 2020; Holtz *et al*., 2015; Ridgley *et al*., 2023). Over three decades, plant cell‐based platforms have been developed, leading to successful cases such as the U.S. FDA‐approved Elelyso® in 2015 and Elfabrio® last year. (Fox, 2012; Hughes *et al*., 2023). However, the plant manufacturing system has rarely been used for the production of therapeutic proteins because plants typically contain plant‐specific glycans that are different from those found in humans (Ma *et al*., 2020). The plant‐specific N‐glycans could induce an immune response, including IgM antibodies when injected into humans, and negatively affect the quality of therapeutic proteins. This is because N‐glycans are important for protein folding, stability, and function, specifically due to protein–protein interactions (Castilho *et al*., 2018). Therefore, the humanization of N‐glycan on a plant‐based platform is a prerequisite for the commercialization of plant‐made pharmaceuticals (PMP) (Gomord and Faye, 2004; Kwon *et al*., 2009).

N‐glycosylation is a critical quality trait of biotherapeutic proteins that influences the efficacy, half‐life, and immunogenicity of the drugs (Donini *et al*., 2021). In this study, we developed glycoengineered rice cell lines, called PhytoRice®, which completely eliminated all the plant‐specific sugar moieties using the CRISPR‐Cas9 technique (Figure 1c). Although efforts to eliminate plant‐specific N‐glycans have continued, the modification of core glycans in α1,3‐FucT and β1,2‐XylT genes has been the primary focus (Cox *et al*., 2006; Göritzer *et al*., 2022; Hanania *et al*., 2017; Herman *et al*., 2023; Jung *et al*., 2021; Mercx *et al*., 2017; Shin *et al*., 2011; Stelter *et al*., 2020; Strasser *et al*., 2008). However, these efforts have not been sufficient to completely remove the plant‐specific N‐glycans such as Lewis a epitope and paucimannosidic N‐glycans (Jung *et al*., 2021). The complete removal of plant‐specific N‐glycans in polyploid plants, such as *Nicotiana* species, has not been reported. In this study, no N‐glycans with β1,3‐galactose or α1,4‐fucose residues were found, despite the low gene editing efficiency of *α1,4‐FucT* in both PMC cell lines (7% and 16% respectively, Figure 1b). This suggests that the function of α1,4‐FucT may be dependent on that of β1,3‐GalT. Consistently, it has been reported that the knockout of *β1,3‐GalT* leads to the inhibition of Lewis a epitope formation (Jung and Kim, 2023).

PhytoRice® was used to successfully produce TMab in this study. Efforts to establish plant‐based monoclonal antibody (mAb) production have continued since the initial report of mAb production in plants (Arya *et al*., 2021; Hiatt *et al*., 1989). Among these efforts, two research groups have presented characterizations of TMab produced in *Nicotiana benthamiana* using a transient expression system (Grohs *et al*., 2010; Komarova *et al*., 2011; McLean, 2017). The plant‐produced TMab (PMT) showed similar HER2 binding affinity and tumour‐killing effectiveness to a commercial TMab in *in vitro* anti‐proliferation assays performed on HER2‐positive breast cancer cell lines. We also demonstrated that wild‐type rice‐based O‐TMab and P‐TMab inhibited the proliferation of HER2+ breast tumour cells by blocking HER2 signalling (Figure 5 and Figure S3). These findings collectively support the idea that a plant manufacturing system could be a feasible alternative for producing therapeutic mAbs with efficacy similar to animal‐based counterparts. Notably, Komarova *et al*. (2011) reported that PMT more effectively inhibited the growth of xenografted tumours derived from human ovarian SKOV3 cancer cells than TMab, although the mechanism was not clear.

The effector function of IgG1 mAbs is significantly influenced by the N‐glycan structure in the Fc region (Pereira *et al*., 2018). The G0 type N‐glycan was predominantly identified in P‐TMab (Figure 4). The removal or reduction of fucosylation on mAbs has been the primary focus on biopharmaceutical glycoengineering. Studies on mAbs have shown that core fucosylation of the N‐glycan in the Fc region affects ADCC function (Shields *et al*., 2002; Umaña *et al*., 1999; Yamane‐Ohnuki *et al*., 2004). The FcγRIIIa gene is known to have two common alleles that encode two variants differing at position 158: Val (V158) or Phe (F158). Among the two variants, FcγRIIIa‐V158 has a higher affinity for human IgG1 (Koene *et al*., 1997; Pereira *et al*., 2018). The V/V FcγRIIIa allotype is known to be present ~12% of the population (Musolino *et al*., 2022). Thus, patients with V/F or F/F allotypes exhibited poor ADCC effectiveness. P‐TMab significantly increased the binding affinity to both FcγRIIIa allotypes, thereby enhancing the *in vitro* ADCC function (Figure 6). Compared to the predominant afucosylated G0 type glycan found in P‐TMab, obinutuzumab produced from the GlycoMab® platform of GlycArt (now merged with Roche) yielded an afucosylated fraction of 30%–50% (Mössner *et al*., 2010; Pereira *et al*., 2018). This mAb is the first Fc‐glycoengineered anti‐CD20 mAb approved by the FDA. Other afucosylated mAbs approved by the FDA include mogamulizumab and benralizumab, which are produced from the Potelligent® platform. This platform has also demonstrated improved ADCC efficacies through Fc‐afucosylation (Duvic *et al*., 2015; Kolbeck *et al*., 2010). In the future, deliberate modification of therapeutic protein glycosylation is expected to become more prevalent due to glycoengineering strategies (Dammen‐Brower *et al*., 2022). Specific glycoengineered platforms capable of attaching unique N‐glycans such as G1 or G0F could be utilized for specific purposes related to Fc effector function and stability.

P‐TMab displayed less liver uptake than TMab (Figure 7), which might be due to the homogeneous N‐glycan type (mainly G0 type) in the Fc region of P‐TMab. The asialoglycoprotein receptor and mannose receptor in hepatocytes are known to respectively recognize terminal galactose and mannose N‐glycan forms attached to glycoproteins, causing rapid clearance (Svecla *et al*., 2023; Wright and Morrison, 1994). Furthermore, a pharmacokinetic study on N‐glycan types of IgG in mouse showed that degalactosylated IgG with terminal GlcNAc has a significantly longer half‐life (Newkirk *et al*., 1996). These indicate that the functions and *in vivo* fate of glycoproteins are significantly affected by the N‐glycan structure. Therefore, we suggest that the PhytoRice® platform could be a superior therapeutic platform for producing IgG1, enhancing ADCC efficacy and half‐life. Currently, there is no data demonstrating the superiority of P‐TMab in terms of *in vivo* inhibitory effects against cancer cells. To further verify this possibility, the efficacies of P‐TMab and TMab should be compared *in vivo*. We plan to examine these *in vivo* efficacies in the near future.

In terms of productivity, our PhytoRice®‐based TMab production reached 91.5 mg/L. Recently, the production of GFP from Hulk cells developed in BY‐2 cells exceeded 1 g/L (Häkkinen *et al*., 2018). This cell line was developed through repeated selection of the original transgenic cell lines, reflecting a more focused effort to isolate high‐production cells, which is promising. Given that high‐productivity CHO cells were only capable of producing minimal amounts of recombinant proteins during their initial development, it is anticipated that the productivity of current plant‐based recombinant proteins can be enhanced even further. CHO cell engineering has been evolving for over six decades since it was established by Theodore Puck in 1956. Modifications to medium composition, culture systems, processing, and genetic manipulation have been implemented to overcome yield issues (Wurm, 2004). It indicates that a plant cell‐based platform could also increase productivity in the near future. Recently, it has been reported that the knock‐in technology was used to precisely insert GFP behind the endogenous *RAmy3D* promoter in rice, aiming to increase productivity in the sugar starvation‐inducible system (Nguyen *et al*., 2022). The application of targeted integration strategy could lead to innovation and increased productivity in the plant cell platform in the near future.

## Materials and Methods

### Plant material and growth condition

The Rice Dongjin (DJ) cultivar and glycoengineered PhytoRice® were utilized for *Agrobacterium*‐mediated transformation. Rice mature seeds, sterilized with 1.5% NaOCl for 40 min, were grown on 2N6 media containing 2 mg/L 2,4‐D at 28 °C for 3‐4 weeks under dark conditions. The formed calli were then used as materials for the transformation to construct PhytoRice®. Selected callus lines were maintained by subculturing every 2‐3 weeks in dark conditions at 28 °C.

### Design of target sequences and vector constructions

To knock out eight genes (*β1,2‐XylT*, *α1,3‐FucT*, *β1,3‐GalT*, *α1,4‐FucT* and four putative hexosaminidase genes) involved in N‐glycosylation, one specific target sequence per gene was selected using the CRISPR‐P gRNA design tool (Liu *et al*., 2017). The information about the selected target sequences and target genes is summarized in Table S1. Polycistronic gRNA sequences, including eight target sequences, were aligned as previously described (Xie *et al*., 2015), and two rice U6 promoters were inserted to drive four gRNAs each (Table S1). The multiplex gRNA sequences, which include *Hin*dIII/*Xba*I recognition sequences at their respective 5' and 3'‐ends, were synthesized by GenScripts (Piscataway, NJ). The synthesized multiplex gRNA sequences were inserted into the pSB11‐Cas9‐HPT vector after digestion with *Hin*dIII/*Xba*I (Figure 1a). The engineered vector, pPM101, was introduced into *Agrobacterium tumefaciens* (LBA4404) using the triparental mating method (Wise *et al*., 2006).

To construct the TMab expression vector, the sequences encoding the light chain (LC) and heavy chain (HC) were fused with the *RAmy3E* gene signal peptide (MGKHHVTLCCVVFAVLCLASSLAQA). These sequences were codon‐optimized for expression in rice and synthesized by GeneArt® (Thermo Fisher Scientific, Waltham, MA). Both pEAQ‐HT vector (Sainsbury and Lomonossoff, 2008) and pMyn75 vector containing the RAmy3D promoter were used to construct constitutive or inducible TMab expression vectors. For cloning of the constitutive TMab expression vector, each gene propagated by PCR was digested with *Pac*I/*Sbf*I and *Sbf*I/*Asc*I, respectively, and then introduced into pEAQ‐HT vector. The pEAQ‐HT‐TMab vector, named pSK446, was used for transformation of rice wild‐type to produce O‐TMab. To make the inducible TMab vector, PCR‐amplified full‐length sequences of TMab heavy chain and light chain genes were digested with SalI/SacI. Subsequently, the digested fragments were individually inserted into the pMyn75 vector, which contains the *RAmy3D* gene promoter‐driven expression system, to create the pMyn75‐TMab LC and pMyn75‐TMab HC vectors. To make a co‐expression vector, the LC expression cassette (pro*RAmy3D*::TMab‐LC::Ter*RAmy3D*) was amplified by PCR and then inserted into the pMyn75‐TMab HC vector after digestion with *Eco*RI, resulting in the formation of pMyn75‐TMab. After digestion with *Hin*dIII, the fragment containing the LC and HC expression cassettes from pMyn75‐TMab was purified and subsequently inserted into the pCleanB vector, which contains the phosphinothricin (PPT)‐resistant gene. This resulting construct was named the pPM102 vector (Figure 2a).

### Plant transformation and selection

Rice seeds were sterilized with 70% ethanol and 1.5% sodium hypochlorite, and then placed onto 2N6 media containing 2 mg/L 2,4‐D (callus‐inducing media, CIM) to induce callus formation from the scutellum. Using embryogenic calli derived from the scutellum or PhytoRice®, *Agrobacterium*‐mediated transformation was performed as previously described (Hiei and Komari, 2008; Kumar *et al*., 2017). The transgenic calli were selected on CIM containing 40 mg/L of hygromycin for pPM101, 15 mg/L of PPT for pPM102 and 50 mg/L of G418 for pSK446. The subculture was conducted every two weeks.

### Polymerase chain reaction (PCR)

To analyse the genome editing patterns, PCR was performed around each target region using genomic DNA extracted from the calli. Each primer set was designed to include a 400‐500 bp‐long target region. The PCR products eluted using the Expin PCR SV mini clean‐up kit (GeneAll, Seoul, South Korea) were sequenced using the Sanger sequencing method. To screen TMab‐expressing lines, PCR was performed using the TMab LC primer set after extracting genomic DNA from PPT‐resistant calli using a modified CTAB method (Kim *et al*., 2011). The primer sets used for PCR and detailed PCR conditions are summarized in Table S6.

### Analysis of genome editing efficiency

Using the sequencing results, we analysed INDELs in the target regions using the Inference of CRISPR Edits (ICE) analysis tool (https://ice.synthego.com/#/) (Hsiau *et al*., 2019).

### Immunoblotting

To investigate the expression of P‐TMab in the selected lines, calli (approximately 100 mg) that had been incubated for 7 days on induction media without sucrose were ground in 1 volume of PBS buffer (pH 7.4) containing 1× cOmplete (Roche, Indianapolis, IN). After centrifuging cell lysates at 16 800 *g* at 4 °C for 10 min, the obtained samples were run on a 7.5% SDS/PAGE gel. The separated proteins were then transferred to a nitrocellulose membrane using the Trans‐blot Turbo Transfer System (Bio‐Rad, Hercules, CA). Interaction with anti‐human IgG‐HRP (1 : 5000; Invitrogen, Waltham, MA) was performed for 30 min after the blocking step using a 5% skim milk‐PBST solution. The target protein was detected using ImageQuant™ LAS 500 (Cytiva, Marlborough, MA) following ECL treatment.

To investigate P‐TMab expression in the selected lines, calli (about 100 mg) 7‐day‐incubated on induction media subtracting sucrose were ground in 1 volume of PBS buffer (pH 7.4) including 1 × cOmplete (Roche, Indianapolis, IN). After cell lysates obtained by centrifugation at 16 800 *g* at 4 °C for 10 min were run in 7.5% SDS/PAGE gel, the separated proteins were transferred into nitrocellulose membrane using Trans‐blot Turbo Transfer System (Bio‐Rad, Hercules, CA). Interaction with anti‐human IgG‐HRP diluted 1 : 5000 (Invitrogen, Waltham, MA) was performed for 30 min after blocking step with 5% skim milk‐PBST solution. The target protein via ECL treatment was detected using ImageQuant™ LAS 500 (Cytiva, Marlborough, MA).

### P‐TMab and O‐TMab production in rice suspension cell culture

Calli were finally selected from the P‐TMab‐expressing line and inoculated in N6 liquid media containing 2 mg/L 2,4‐D and 0.02 mg/L kinetin. The cultures were then cultivated at 28 °C with shaking at 110 rpm. The suspension culture was subcultured every 7 days by adding one volume of inoculum to four volumes of N6 liquid media. The established suspension cells were used to produce P‐TMab and O‐TMab. For inducing P‐TMab, Suspension cells were harvested by centrifugation and then inoculated into N6 liquid media without sucrose (induction liquid media) to achieve a 50% (V/V) cell density. The cells were then incubated at 28 °C with shaking at 110 rpm for 7 days. O‐TMab was directly obtained from 8‐day‐cultured media and cells without induction phase (Figure S3d).

### Enzyme‐linked immunosorbent assay (ELISA)

Both cells and spent media were harvested 5 days after P‐TMab induction. Cell lysates were then prepared using 1xPBS (pH 7.4) containing 1× cOmplete protease inhibitor cocktail (Roche, Indianapolis, IN). The quantity of P‐TMab was measured using the Human IgG total ELISA kit (Invitrogen, Waltham). For the assay, all samples were diluted at a ratio of 1 : 20 000. Then, 100 μL of each sample and 50 μL of horseradish peroxidase (HRP)‐conjugated anti‐human total IgG monoclonal antibody, diluted at a ratio of 1 : 100, were added to the microwell plates coated with monoclonal antibody to human total IgG. After incubating for 1 h at room temperature, the colour reaction was carried out using tetramethylbenzidine for 30 min. The absorbance of the samples at 450 nm was measured using a microplate reader. A standard curve was generated using a 5‐parameter curve fit in SoftMax Pro 7.1, and the quantities of P‐TMab were subsequently calculated. All samples were analysed in both biological and technical duplicates.

### Antibody purification

The spent media was filtered using a Miracloth (Merck Life Sciences, Darmstadt, Germany) and then passed through a 0.2 μm depth filter. This was followed by ultrafiltration using Vivaflow (50 MWCO, Sartorius, Göttingen, Germany). P‐TMab and O‐TMab were purified according to the method described previously (Ma *et al*., 2015). The quantity of purified TMab was determined using NanoDrop (Thermo Fisher Scientific, Waltham, MA), and its quality was assessed using SDS‐Polyacrylamide gel electrophoresis.

### Full‐length amino acid sequencing analysis

P‐TMab and TMab (Herceptin®; purchased from Roche, Basel, Switzerland) were subjected to trypsin or Glu‐C treatment under reducing conditions. The cleaved peptide was separated using a reverse‐phase column (C18) via RP‐UPLC. Mass spectrometry data of peptide peaks separated on UPLC was obtained through MS1 analysis, and the molecular weight of the peptide determined from the MS data identified it as a peptide with the expected sequence. The peptide's amino acid sequence was determined through analysis of the fragmentation spectrum. After fragmenting the peptides, information from the UPLC‐MS1 analysis was used to identify the peptide peaks and determine the sequence information of the peptides. The b‐ions and y‐ions, cut from the N‐terminal and C‐terminal regions, respectively, were generated by fragmentation through ESI (Q‐TOF). The amino acid sequence was obtained by analysing the masses of b‐ions and y‐ions.

### N‐glycan structure analysis using MALDI‐TOF

To analyse the N‐glycan pattern of secreted proteins, the spent media harvested from a 2‐week suspension culture was filtered through a 0.2 μm pore‐sized membrane and then concentrated using a 5 kDa cut‐off Vivaspin (Sartorius, Göttingen, Germany). The total secreted proteins or purified TMab (200 μg) were digested with 20 μg of trypsin in a reaction buffer (100 mm Tris–HCl, pH 8.2, 1 mm CaCl2) at 37 °C for 16 h. The trypsin‐digested peptides were desalted using a reverse‐phase Sep‐Pak C18 cartridge (Restek, Centre County, PA) and then lyophilized. The glycopeptides were resuspended in a 50 mm sodium phosphate buffer (pH 5.2) and then incubated with 1 U of peptide‐N‐glycosidase A (New England Biolabs, Ipswich, MA) at 37 °C for 18 h. The N‐linked glycans released were purified from the peptides using a Sep‐Pak C18 (Restek, Centre County, PA) with a 5% acetic acid elution solution. The samples were lyophilized and then permethylated. The permethylated glycans were subsequently purified using a Sep‐Pak C18 (Restek, Centre County, PA) and then freeze‐dried for analysis by matrix‐assisted laser desorption/ionization time‐of‐flight mass spectrometry (MALDI‐TOF MS).

### N‐glycan structure analysis using UPLC‐FLD method

The deglycosylation of P‐TMab and TMab, N‐glycan labelling, and purification were performed using the Glycoworks RapiFluor‐MS N‐Glycan Kit (Waters, Milford, MA), following the manufacturer's instructions. The intact mAb Mass Check Standard (186008843; Waters, Milford, MA) was used as the control standard. For the separation of the labelled N‐glycan, the Ultra Performance Liquid Chromatograph (UPLC I‐class system; Waters, Milford, MA) was used with the ACQUITY UPLC Glycan BEH amide (130 Å, 1.7 μm, 2.1 × 150 mm; Waters, Milford, MA) column and the fluorescence detector. The following gradient conditions were used (mobile phase A, 50 mm ammonium formate solution, pH 4.4; mobile phase B, 100% acetonitrile). The gradient profile was as follows: 0–35 min, 75% to 54% mobile phase B, flow rate of 0.4 mL/min; 35‐36.5 min, 54% to 0% B, 0.4 to 0.2 mL/min; 36.5–39.5 min, 0% B, 0.2 mL/min; 39.5‐43.1 min, 0% to 75% B, 0.2 mL/min; 43.1‐47.6 min, 75% B, 0.2 to 0.4 mL/min; 47.6–55 min, 75% B, 0.4 mL/min. The column oven temperature was maintained at 60 °C, and the sample injection volume was 10 μL. Fluorometric detection was performed using excitation and emission wavelengths of 265 nm and 425 nm, respectively. The molecular mass of the labelled N‐Glycan was determined using the Orbitrap mass spectrometer (LTQ Orbitrap; Thermo Fisher Scientific, San Jose, CA) equipped with the UPLC system (UltiMate™ 3000; Thermo Fisher Scientific, San Jose, CA).

### Surface plasmon resonance (SPR)

The binding of TMab and P‐TMab to human FcγRIIIa variants (V158 and F158 allotypes) (10389‐H08H1 and 10389‐H08H; Sino Biological, Beijing, China) was assessed using a Biacore X100 surface plasmon resonance biosensor (Cytiva, Marlborough, MA). CM5 chips were covalently coupled with *S. aureus* Protein A (Merck P6031; Merck Life Sciences, Darmstadt, Germany) to achieve a response level of 3000 RU. In a standard assay, 750‐1000 RU of antibodies were immobilized on the active channel (Fc2), and the receptor (FcγRIIIa 25‐800 nM) was flowed over both channels at a rate of 30 μL/min. The resulting data were fitted using a Langmuir 1 : 1 model (BIAEvaluation v.3.2; Cytiva, Marlborough, MA).

### Bio‐layer interferometry (BLI)

The binding affinity of TMab, P‐TMab or O‐TMab with human HER2 (10004‐H08H; Sino Biological, Beijing, China) was measured using Octet HTX based on BLI (ForteBio, Fremont, CA). Each TMab (10 μg/mL) was captured by anti‐human Fc capture (AHC) sensor (18‐5064; ForteBio, Fremont, CA), and the kinetics of binding with human HER2 were subsequently analysed. The concentration of utilized HER2 ranged from 12.5 nM to 0.78 nM, achieved through serial dilution (1/2 dilution).

### *In vitro* anti‐proliferation assay

BT‐474 cells were seeded at 4⨯10^3^ cells per well in a 96‐well plate. Cells were treated with TMab, P‐TMab or O‐TMab at various concentrations and then incubated for 72 h. After adding CCK‐8 solution (Dojindo, Rockville, MD), the cells were further incubated for 2 h. The absorbance was measured at 450 nm using a microplate reader (ThermoFisher Scientific, Hampton, NH). Samples were processed in three biological replicate.

### ADCC assay

The HER2‐positive cell line, BT‐474 cells, was seeded at a density of 1.25×10^4^ cells per well in a 96‐well plate and then incubated in a CO_2_ incubator for 24 h. ADCC buffer containing 4% Low IgG serum (Promega, Madison, MI), was treated with TMab, P‐TMab or O‐TMab antibodies at various concentrations. Jurkat cells were treated at a concentration of 3×10^6^ cells/mL and then incubated for 24 h. After the incubation, Bio‐Glo reagent (Promega, Madison, MI) was added, and luminescence was measured using a Luminoskan (Thermo Scientific, Waltham, MA). Samples were processed in three biological replicates.

### Mouse xenograft tumour model and nuclear medicine imaging

Female nude mice (6–8 weeks old) were obtained from Orient Bio (Sungnam, South Korea) and acclimated for 1 week. All mice were housed in specific‐pathogen‐free conditions at a controlled temperature of 23 ± 2 °C with a 12‐h light/dark cycle. Animal experiments were conducted following ethical guidelines and approved by the Institutional Animal Care and Use Committee of Seoul National University Hospital. To promote BT‐474 breast cancer tumour cells in nude mice, oestrogen supplementation is necessary. Therefore, mice were subcutaneously injected with oestradiol pellets that released 2.5 mg of oestrogen for 60 days (Innovative Research of America, Sarasota, FL) into the dorsal cervicis before tumour inoculation. BT‐474 cells (2×10^7 cells) were subcutaneously injected into the right flank of a mouse.

For conjugation with radioactive isotopes, antibodies were reduced by adding 1 mm of tris(2‐carboxyethyl)phosphine (TCEP) for 2 h at 37 °C, then 20 nmol/μL of DBCO‐PEG4‐maleimide (dibenzocyclooctyne‐PEG4‐maleimide) was added and incubated at room temperature for 2 h. In this study, 18 nmol of 3‐azidopropyl‐NOTA (3‐azidopropyl‐1,4,7‐triaza‐cyclononane‐1,4,7‐triacetic acid) was added to ^111^InCl_3_ and the reaction mixture was incubated at 70 °C for 10 min in a heating block. Subsequently, 3‐azidopropyl‐NOTA‐^111^In was added to DBCO‐antibodies for 1 h at 37 °C in a heating block. The labelling efficiency at each step was determined using Instant Thin Layer Chromatography (ITLC‐SG). Strips were counted with a Bio‐Scan AR‐2000 System Imaging Scanner (Bio‐Scan Inc., Washington DC).

^111^In‐labelled TMab or P‐TMab antibody (7.34 MBq ± 0.758) was administered by tail vein injection to BT‐474 tumour‐bearing mice and SPECT/CT images were acquired using nanoSPECT plus (Mediso, Budapest, Hungary) at 0, 6, 24, and 48 h post‐injection.

### Data analysis

The data in this study are depicted as mean values from three biological replicates, accompanied by standard errors of the mean (SEM) indicated as ±. Statistical significance was assessed through unpaired Student's *t*‐tests (**P < 0.01; ****P < 0.0001).

## Funding

This research was supported by the National Research Foundation of Korea (NRF) grants funded by the Korean government (MEST) (No. 2020R1A2C1014133 for Seong‐Ryong Kim; No. NRF‐2020R1A2C2011695 for Hyewon Youn). Julian Ma received generous support from the Sir Joseph Hotung Charitable Trust; the European Union's Horizon 2020 programme under Grant Agreements 774078 (Pharma‐Factory) and 760331 (Newcotiana), and the BBSRC (Celfacto project).

## Conflict of interest

All authors declare no competing interests.

## Author contributions

S‐RK and HY conceived and designed this study supervised the work and finalized the manuscript. J‐HS conducted the experimental design, vector construction, establishment of cell lines, protein purification, data analysis and writing the manuscript. SO conducted *in vitro* anti‐proliferation assay and *in vivo* distribution, and contributed to writing manuscript. M‐HJ conducted plant transformation and established cell lines. S‐YL conducted an *in vitro* ADCC assay. CM and H‐BO contributed to N‐glycan analysis and data analysis. Y‐JE contributed to protein purification and characterization of P‐TMab. HB screened and analysed mutants. J‐CK and GRL contributed to *in vitro* anti‐proliferation assay and data analysis. MJP and JK‐CM contributed to protein binding affinity assay and data analysis. H‐SG contributed to data analysis. All authors read and approved the final manuscript.

## Supporting information


**Figure S1** Investigation of genome editing of callus line #1‐12‐20‐11. (a) Editing efficiency was analysed using Synthego program. T‐DNA region of pPM101 was integrated into rice genome via Agrobacterium‐mediated transformation method. Among hygromycine resistant‐calli, #1‐12‐20‐11 line was finally selected via examining INDEL efficiency. The efficiency of genome editing was analysed by ICE tool (https://ice.synthego.com/#/). (b) Immunoblotting of #1‐12‐20‐11 line using anti‐β1,2‐XylT and anti‐α1,3‐FucT. Total cellular proteins (10 μg) from the #1‐12‐20‐11 line and WT callus separated in 12% SDS‐PAGE gel were stained using Coomassie blue (left panel), or transferred to nitrocellulose membranes for immunoblotting using either anti‐β1,2‐xylose (middle panel) or anti‐α1,3‐fucose (right panel). M, size marker; Lane 1, wild‐type (Dongjin); Lane 2, # 1‐12‐20‐11. (c) Images of #1‐12‐20‐11 callus (top panel) and WT callus (bottom panel). Bar = 1 cm.
**Figure S2** Summary of the T‐DNA insertion into rice genome of PMC1 (a) and PMC2 (b). Inverse PCR (IPCR) was performed to identify the T‐DNA location in the genome of each cell. (a) T‐DNA was found to be integrated within LOC_Os01g29409. (b) T‐DNAs in the PMC2 genome were identified in LOC_Os01g29409 and the intergenic region between LOC_Os02g44780 and LOC_Os02g44810.
**Figure S3** Construction of constitutive TMab expression vector and screening of O‐TMab‐expressed callus lines from *Agrobacterium*‐mediated transgenic wild‐type (non‐glycoengineered) rice calli. (a) The diagram of T‐DNA region of pSK446 expressing TMab. Codon‐optimized TMab light chain (TMab_LC) and heavy chain (TMab_HC) genes were inserted into separate expression cassettes driven by the cauliflower mosaic virus (CaMV) 35S promoter and then introduced into the pEAQ‐HT vector, resulting in the construction of the TMab LC and HC co‐expression vector, pSK446. Both 5′ and 3′ UTR sequences are from RNA‐2 in cowpea mosaic virus (CPMV) genome. Tnos, nopaline synthase gene terminator; *npt*, neomycin phosphotransferase gene; RB, right border; LB, left border. (b) gDNA PCR using the primer set for the TMab‐LC gene. Approximately 700 base pair‐sized bands were detected in G418‐resistant callus lines. NC, non‐transgenic WT callus; PC, pSK446 plasmid. (c) RT‐PCR of the callus lines selected by gDNA PCR. After cDNA was synthesized from total RNA (0.5 μg) using oligo‐dT primer, PCR was performed using *TMab‐HC*, *TMab‐LC* and *OsUbi* gene primer. (d) O‐TMab expression pattern observed in line #44. The suspension cell culture was established via the inoculation of #44 calli. After subculture, the O‐TMab expression was examined in the cultured media from Day 0 (D0) to Day 11 (D11) using immunoblotting. The harvested media (10 μL) was separated under non‐reducing conditions in 6% SDS/PAGE. The O‐TMab was detected using rabbit α‐human IgG‐HRP at a dilution factor of 1 : 5000. The arrowhead indicates a TMab. PC, TMab (5 ng were loaded).
**Figure S4** *In vitro* ADCC assay of O‐TMab and TMab. The HER2‐positive BT‐474 cells seeded in a 96‐well plate (a density of 1.25×10^4^ cells) were incubated in a CO_2_ incubator for 24 hours. ADCC buffer treated with TMab, or O‐TMab at various concentrations and Jurkat cells were treated at a concentration of 3 × 10^6^ cells/mL and then incubated for 24 hours. The data in this study are depicted as mean values from three biological replicates, accompanied by standard errors of the mean (SEM) indicated as ±.
**Table S1** Information of sgRNAs for CRISPR‐cas9‐based knock‐out of eight target genes involved in plant‐specific N‐glycosylation in rice.
**Table S2** The relative amount of N‐glycan in PMCs.
**Table S3** Analysis of full‐length amino acid sequence of P‐TMab by LC/MS.
**Table S4** Analysis of full‐length amino acid sequence of P‐TMab by LC/MS/MS.
**Table S5** The relative amount of N‐glycan in P‐TMab and TMab.
**Table S6** List and sequences of Primers used in this study.

## Data Availability

The data that support the findings of this study are available on request from the corresponding author. The data are not publicly available due to privacy or ethical restrictions.
