# Supplementary material for: Enhanced efficacy of glycoengineered rice cell‐produced trastuzumab
Source: Plant Biotechnol J. 2024 Jul 17;22(11):3068–81. doi: 10.1111/pbi.14429 (PMC11500988; doi:10.1111/pbi.14429)
Supplement: Supplementary file 1 — Figure S1 Investigation of genome editing of callus line #1‐12‐20‐11. (a) Editing efficiency was analysed using Synthego program. T‐DNA region of pPM101 was integrated into rice genome via Agrobacterium‐mediated transformation method. Among hygromycine resistant‐calli, #1‐12‐20‐11 line was finally selected via examining INDEL efficiency. The efficiency of genome editing was analysed by ICE tool (https://ice.synthego.com/#/). (b) Immunoblotting of #1‐12‐20‐11 line using anti‐β1,2‐XylT and anti‐α1,3‐FucT. Total cellular proteins (10 μg) from the #1‐12‐20‐11 line and WT callus separated in 12% SDS‐PAGE gel were stained using Coomassie blue (left panel), or transferred to nitrocellulose membranes for immunoblotting using either anti‐β1,2‐xylose (middle panel) or anti‐α1,3‐fucose (right panel). M, size marker; Lane 1, wild‐type (Dongjin); Lane 2, # 1‐12‐20‐11. (c) Images of #1‐12‐20‐11 callus (top panel) and WT callus (bottom panel). Bar = 1 cm. Figure S2 Summary of the T‐DNA insertion into rice genome of PMC1 (a) and PMC2 (b). Inverse PCR (IPCR) was performed to identify the T‐DNA location in the genome of each cell. (a) T‐DNA was found to be integrated within LOC_Os01g29409. (b) T‐DNAs in the PMC2 genome were identified in LOC_Os01g29409 and the intergenic region between LOC_Os02g44780 and LOC_Os02g44810. Figure S3 Construction of constitutive TMab expression vector and screening of O‐TMab‐expressed callus lines from Agrobacterium‐mediated transgenic wild‐type (non‐glycoengineered) rice calli. (a) The diagram of T‐DNA region of pSK446 expressing TMab. Codon‐optimized TMab light chain (TMab_LC) and heavy chain (TMab_HC) genes were inserted into separate expression cassettes driven by the cauliflower mosaic virus (CaMV) 35S promoter and then introduced into the pEAQ‐HT vector, resulting in the construction of the TMab LC and HC co‐expression vector, pSK446. Both 5′ and 3′ UTR sequences are from RNA‐2 in cowpea mosaic virus (CPMV) genome. Tnos, nopaline synthase gene term [file PBI-22-3068-s001.docx]

**Supporting Information for**

**Enhanced Efficacy of Glyco-Engineered Rice Cell-Produced Trastuzumab**

Jun-Hye Shin^1,2^, Sera Oh^3,4^, Mi-Hwa Jang^2^, Seok-Yong Lee^3,4^, Chanhong Min^5^, Young-Jae Eu^2^, Hilal Begum^1^, Jong-Chan Kim^1^, Gap Ryol Lee^1^, Han-Bin Oh^5^, Matthew J. Paul ^6^, Julian K-C Ma^6^, Ho-Shin Gwak^7^, Hyewon Youn^3,4^*, Seong-Ryong Kim^1,2^*

^1^Department of Life Science, Sogang University, Seoul, Republic of Korea

^2^PhytoMab Co. Ltd., South Korea

^3^Department of Nuclear Medicine, Cancer Imaging Center, Seoul National University Hospital, Seoul, South Korea

^4^Cancer Research Institute, Seoul National University College of Medicine, Seoul, South Korea

^5^Department of Chemistry, Sogang University, Seoul, South Korea

^6^Hotung Molecular Immunology Unit, Institute for Infection & Immunity, St George's University of London, Cranmer Terrace, London, SW17 0RE, United Kingdom

^7^National Cancer Center Korea, Goyang-si, Kyunggi-do, South Korea

**Supporting information: Figure S1~S4; Table S1~S6**


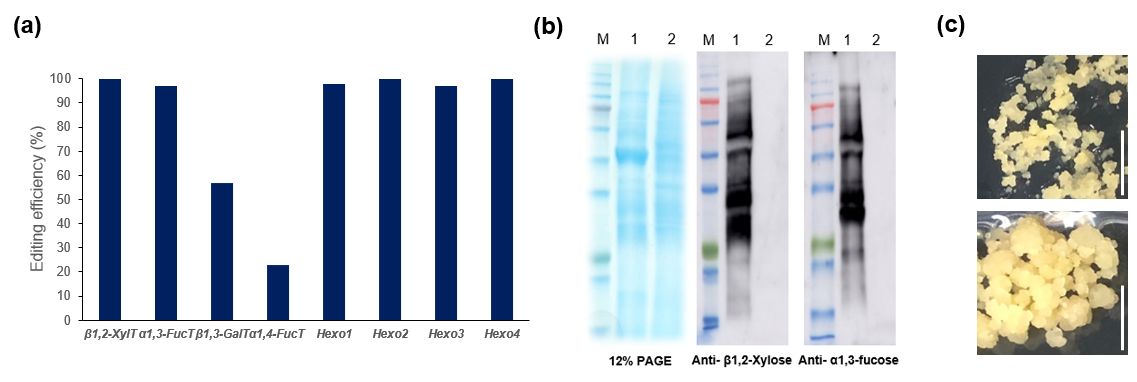


**Figure S1.** Investigation of genome editing of callus line #1-12-20-11. (a) Editing efficiency was analyzed using Synthego program. T-DNA region of pPM101 was integrated into rice genome via Agrobacterium-mediated transformation method. Among hygromycine resistant-calli, #1-12-20-11 line was finally selected via examining INDEL efficiency. The efficiency of genome-editing was analyzed by ICE tool (https://ice.synthego.com/#/). (b) Immunoblotting of #1-12-20-11 line using anti-β1,2-XylT and anti-α1,3-FucT. Total cellular proteins (10 μg) from the #1-12-20-11 line and WT callus separated in 12% SDS-PAGE gel were stained using Coomassie blue (left panel) or transferred to nitrocellulose membranes for immunoblotting using either anti-β1,2-xylose (middle panel) or anti-α1,3-fucose (right panel). M, size marker; Lane 1, Wild-type (Dongjin); Lane 2, # 1-12-20-11. (c) Images of #1-12-20-11 callus (top panel) and WT callus (bottom panel). Bar = 1 cm.


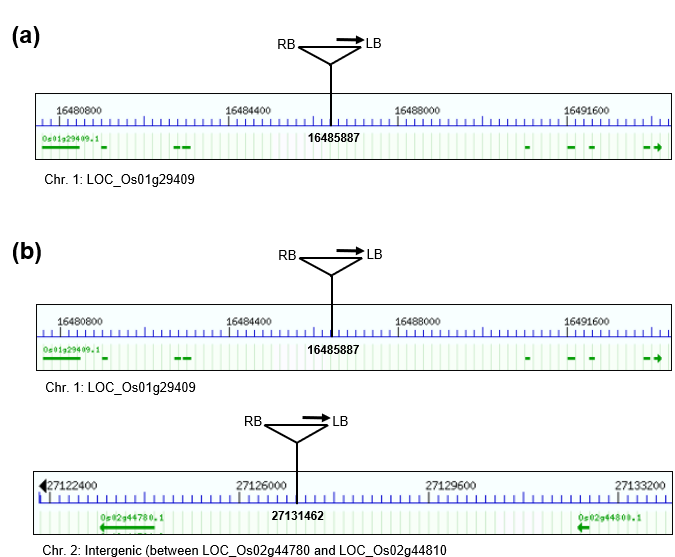


**Figure S2.** Summary of the T-DNA insertion into rice genome of PMC1 (a) and PMC2 (b). Inverse PCR (IPCR) was performed for identify the T-DNA location in the genome of each cell. (a) T-DNA was found to be integrated within LOC_Os01g29409. (b) T-DNAs in PMC2 genome were identified in LOC_Os01g29409 and the intergenic region between LOC_Os02g44780 and LOC_Os02g44810.


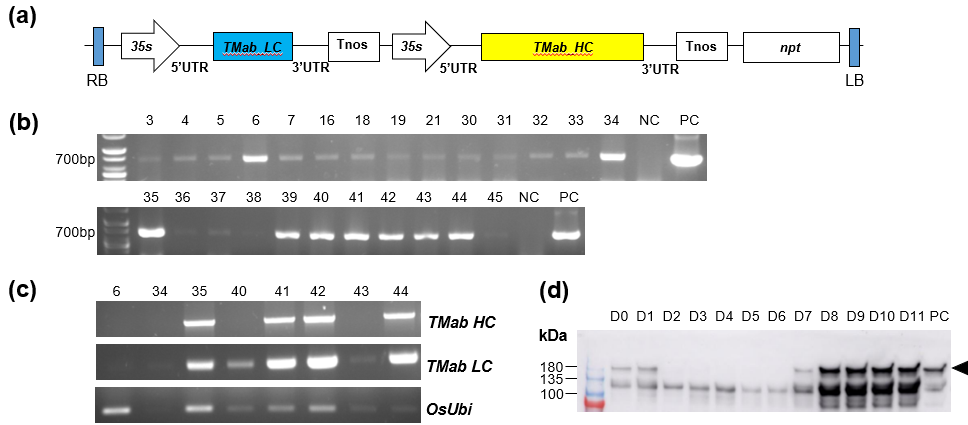


**Figure S3.** Construction of constitutive TMab expression vector and screening of O-TMab-expressed callus lines from Agrobacterium-mediated transgenic wild-type (non-glycoengineered) rice calli. (a) The diagram of T-DNA region of pSK446 expressing TMab. Codon-optimized TMab light chain (TMab_LC) and heavy chain (TMab_HC) genes were inserted into separate expression cassettes driven by the cauliflower mosaic virus (CaMV) 35S promoter, and then introduced into pEAQ-HT vector, resulting in the construction of the TMab LC and HC co-expression vector, pSK446. Both 5’ and 3’ UTR sequences are from RNA-2 in cowpea mosaic virus (CPMV) genome. Tnos, nopaline synthase gene terminator; npt, neomycin phosphotransferase gene; RB, right border; LB, left border. (b) gDNA PCR using the primer set for the TMab-LC gene. Approximately 700 base pair-sized bands were detected in G418-resistant callus lines. NC, non-transgenic WT callus; PC, pSK446 plasmid. (c) RT-PCR of the callus lines selected by gDNA PCR. After cDNA was synthesized from total RNA (0.5 µg) using oligo-dT primer, PCR was performed using TMab-HC, TMab-LC and OsUbi gene primer. (d) O-TMab expression pattern observed in lines #44. The suspension cell culture was established via the inoculation of #44 calli. After subculture, the O-TMab expression was examined in the cultured media from day 0 (D0) to Day 11 (D11) using immunoblotting. The harvested media (10 µl) was separated under non-reducing conditions in 6% SDS/PAGE. The O-TMab was detected using rabbit α-human IgG-HRP at a dilution factor 1:5,000. The arrowhead indicates a TMab. PC, TMab (5 ng were loaded).

**Figure S4.** *In vitro* ADCC assay of O-TMab and TMab. The HER2-positive BT-474 cells seeded in a 96-well plate (a density of 1.25×10^4^ cells) were incubated in a CO_2_ incubator for 24 hours. ADCC buffer treated with TMab, or O-TMab at various concentrations and Jurkat cells were treated at a concentration of 3×10^6^ cells/mL and then incubated for 24 hours. The data in this study are depicted as mean values from three biological replicates, accompanied by standard errors of the mean (SEM) indicated as ±.

**Table S1.** Information of sgRNAs for CRISPR-cas9-based knock-out of 8 target genes involved in plant specific N-glycosylation in rice.

| Gene name (Locus number) | Seq | Target region | Direction |
| --- | --- | --- | --- |
| ***β1,2-XylT*** (LOC_Os08g39380) | ACTCCTGTGAGGGGTACTTC | Exon 1 | + |
| ***α1,3-FucT*** (LOC_Os06g12390) | AGAGAGTATCCTCAGATCGA | Exon 2 | + |
| ***α1,4-FucT*** (LOC_Os12g07290) | GTACGGCGCCAACTCGACCG | Exon 1 | + |
| ***β1,3-GalT*** (LOC_Os06g12390) | TCATTCTTCGAATGGAATAT | Exon 1 | - |
| ***Hexo1*** (LOC_Os05g02510) | GCTGCCGAGGAACTTCACCT | Exon 1 | - |
| ***Hexo2*** (LOC_Os03g11980) | GACCGGGTAGAAATTCCTGG | Exon 1 | + |
| ***Hexo3*** (LOC_Os01g66700) | CTTGAAGGATGCCTTCCAGA | Exon 2 | - |
| ***Hexo4*** (LOC_Os05g34320) | AGGGGAGCGTCGTCGAGGTG | Exon 1 | + |

**Table S2.** The relative amount of N-glycan in PMCs

| Type of glycan (%) | WT (Cell) | WT (Media) | PMC1 (Cell) | PMC1 (Media) | PMC2 (Cell) | PMC2 (Media) |
| --- | --- | --- | --- | --- | --- | --- |
| MM | 0 | 0 | 0 | 1 | 0 | 0 |
| MMX | 18.0 | 2.7 | 0 | 0 | 0 | 0 |
| MMXF | 55.8 | 81.6 | 0 | 0 | 0 | 0 |
| GnM | 0 | 0 | 19.2 | 1.6 | 7.7 | 18.1 |
| GnMX | 0.6 | 0 | 0 | 0 | 0 | 0 |
| GnMXF | 7.5 | 11.4 | 0 | 0 | 0 | 0 |
| GnGn | 0.0 | 0 | 80.8 | 97.4 | 92.3 | 81.9 |
| GnGnXF | 3.3 | 1.3 | 0 | 0 | 0 | 0 |
| AGnX | 0.1 | 0 | 0 | 0 | 0 | 0 |
| AGnXF | 4.1 | 2.1 | 0 | 0 | 0 | 0 |
| AGnGnXF | 2.4 | 0.8 | 0 | 0 | 0 | 0 |
| AFGnGnXF | 3.8 | 0 | 0 | 0 | 0 | 0 |
| AAGnGnXF | 1.6 | 0 | 0 | 0 | 0 | 0 |
| AAFFGnGnXF | 1.9 | 0 | 0 | 0 | 0 | 0 |
| Total | 100 | 100 | 100 | 100 | 100 | 100 |

**Table S3.** Analysis of full-length amino acid sequence of P-TMab by LC/MS

| Protein | Chain | Enzyme | No. of identified residue/ No. of total residue | Coverage (%) | Total coverage (%) |
| --- | --- | --- | --- | --- | --- |
| TMab | Light | Trypsin | 211/214 | 98.6 | 100 |
|  |  | Glu-C | 214/214 | 100.0 |  |
|  | Heavy | Trypsin | 450/450 | 100.0 | 100 |
|  |  | Glu-C | 450/450 | 100.0 |  |
| P-TMab | Light | Trypsin | 211/214 | 98.6 | 100 |
|  |  | Glu-C | 214/214 | 100.0 |  |
|  | Heavy | Trypsin | 450/450 | 100.0 | 100 |
|  |  | Glu-C | 450/450 | 100.0 |  |

**Table S4.** Analysis of full-length amino acid sequence of P-TMab by LC/MS/MS.

| Protein | Chain | Enzyme | No. of identified residue/No. of total residue | Coverage (%) | Total coverage (%) | | Sequence homology (%) |
| --- | --- | --- | --- | --- | --- | --- | --- |
| TMab | Light | Trypsin | 192/214 | 89.7 | 94.0 | 100 | |
|  |  | Glu-C | 164/214 | 76.6 |  |  |  |
|  |  | Trypsin+Glu-C | 210/214 | 98.1 |  |  |  |
|  | Heavy | Trypsin | 383/450 | 85.1 |  |  |  |
|  |  | Glu-C | 252/450 | 56.0 |  |  |  |
|  |  | Trypsin+Glu-C | 414/450 | 92.0 |  |  |  |
| P-TMab | Light | Trypsin | 197/214 | 92.1 | 93.7 | 100 | |
|  |  | Glu-C | 157/214 | 73.4 |  |  |  |
|  |  | Trypsin+Glu-C | 210/214 | 98.1 |  |  |  |
|  | Heavy | Trypsin | 365/450 | 81.1 |  |  |  |
|  |  | Glu-C | 275/450 | 61.1 |  |  |  |
|  |  | Trypsin+Glu-C | 412/450 | 91.6 |  |  |  |

**Table S5.** The relative amount of N-glycan in P-TMab and TMab.

| Type of glycan | TMab | P-TMab |
| --- | --- | --- |
| G0-GN | 0.7 | 4.5 |
| G0F-GN | 1.0 | 0.0 |
| G0 | 5.4 | 95.5 |
| G0F | 42.6 | 0.0 |
| G1F-GN | 1.9 | 0.0 |
| G1 | 3.4 | 0.0 |
| G1F | 39.0 | 0.0 |
| G2F | 5.9 | 0.0 |
| Total (%) | 100.0 | 100.0 |

**Table S6.** List and sequences of Primers used in this study.

| Primer name | Sequences (From 5' to 3') | Size | Application |
| --- | --- | --- | --- |
| β1,2-XylT-F | ACAACAGCAACAACCATCGG | 550 bp | Genome editing efficiency |
| β1,2-XylT-R | CATCACCTGGTCGAGCGG |  |  |
| α1,3-FucT-F | CCCTCAAGCTTTATGCTCAACT | 591 bp | Genome editing efficiency |
| α1,3-FucT-R | TGTGTGACTTCTCAACATGGAT |  |  |
| α1,4-FucT-F | CTCCCACCCTTTCCACTGTA | 691 bp | Genome editing efficiency |
| α1,4-FucT-R | ACGTGTACACCCCGTCGAG |  |  |
| β1,3-GalT-F | TGCAGTTCAGAATCCACAGAA | 469 bp | Genome editing efficiency |
| β1,3-GalT-R | AAGCACAATTGGAGGGTCTG |  |  |
| Hexo1-F | ATACCCGGGCACATTTACAG | 483 bp | Genome editing efficiency |
| Hexo1-R | CCACTCCAAGCTCCAGCTAC |  |  |
| Hexo2-F | CGACGAGTCCTACACGCTCT | 369 bp | Genome editing efficiency |
| Hexo2-R | GAGTAGGAGCCGGAGTTGG |  |  |
| Hexo3-F | CATTACGAAATGGCTTTTCCAT | 535 bp | Genome editing efficiency |
| Hexo3-R | TGCTATTCAACAGGCCAAGTTA |  |  |
| Hexo4-F | GGCGTTTCTCTTCATCTTCTTG | 451 bp | Genome editing efficiency |
| Hexo4-R | CAGCTCTATCAGCGTCACCA |  |  |
| TMab-LC-F_SalI | GCGTCGACATGGGCAAGCACCACGTGAC | 723 bp | Cloning and PCR |
| TMab-LC-R_SacI | GCGAGCTCTCATCAGCACTCGCCGCGGT |  |  |
| TMab-HC-F_SalI | GCGTCGACATGGGCAAGCACCACGTGAC | 1431 bp | Cloning |
| TMab-HC-R_SacI | GCGAGCTCTCATCACTTGCCTGGAGACA |  |  |
